# Argonaute 2 promotes myeloma angiogenesis via microRNA dysregulation

**DOI:** 10.1186/1756-8722-7-40

**Published:** 2014-05-07

**Authors:** Shuang Wu, Wenjun Yu, Xiaoyan Qu, Rong Wang, Ji Xu, Qiguo Zhang, Jiaren Xu, Jianyong Li, Lijuan Chen

**Affiliations:** 1 Department of Hematology, First Affiliated Hospital of Nanjing Medical University, Jiangsu Province Hospital, 300 Guangzhou Road, Nanjing 210029, China; 2 Nanjing Gulou Hospital, Nanjing, China

**Keywords:** Argonaute 2, microRNA, let-7 family, miR-17/92 cluster, miR-145, Myeloma, Angiogenesis

## Abstract

**Background:**

Dysregulated microRNA (miRNA) expression contributes to cancer cell proliferation, apoptosis and angiogenesis. Angiogenesis is a hallmark of multiple myeloma (MM) development and progression. Argonaute 2 (AGO2) protein, a core component of the RNA-induced silencing complex (RISC), can directly bind to miRNAs and mediate target messenger RNA (mRNA) degradation. A previous study showed that AGO2 knockdown suppressed human umbilical vein endothelial cell (HUVEC) growth and tube formation. However, the roles and molecular mechanisms of AGO2-induced myeloma angiogenesis are not yet fully understood. The aim of this study was to characterize these roles and effects and their associated mechanisms.

**Results:**

Supernatants from AGO2-overexpressing MM lines induced HUVEC migration and accelerated tube formation. Conversely, supernatants from AGO2-knockdown MM lines suppressed HUVEC cell migration and tube formation. Moreover, a chick chorioallantoic membrane (CAM) assay was used to demonstrate that AGO2 could drive neovessel formation in MM lines *in vivo*. Using an miRNA microarray, we observed that 25 miRNAs were upregulated and 7 were downregulated in response to AGO2. Most let-7 family members and 2 miR-17/92 cluster members (miR-17a and miR-92-1), all known pro-angiogenic miRNAs, were positively regulated by AGO2 whereas anti-angiogenic miRNAs such as miR-145 and miR-361 were negatively regulated by AGO2.

**Conclusions:**

We conclude that AGO2 can drive neovessel formation *in vitro* and *in vivo* by dysregulating the expression of some angiogenic miRNAs. The pro-angiogenic miRNAs of the let-7 family and the miR-17/92 cluster, along with the anti-angiogenic miRNA miR-145, play crucial roles in AGO2-mediated angiogenesis by targeting angiogenesis-related genes.

## Background

Multiple myeloma (MM) is an incurable disease characterized by the clonal proliferation of malignant plasma cells and increased monoclonal immunoglobulin expression, along with bone lesions and renal failure. A variety of chromosomal abnormalities such as translocations, gene mutations and epigenetic alterations are involved in myelomagenesis [1]. In addition to these oncogenic events, interactions between MM cells and the bone marrow microenvironment are well known to play a critical role in MM cell growth, survival, differentiation, migration and chemotherapeutic resistance [2-4]. The tumour microenvironment, which comprises a variety of cell types, can secrete angiogenic cytokines including vascular endothelial growth factor (VEGF), platelet-derived growth factor (PDGF) and fibroblast growth factor (FGF), thus promoting tumour angiogenesis via endothelial cell (EC) activation [5,6]. Angiogenesis, a prominent feature of MM, can predict the prognosis of MM patients and is a hallmark of MM development and progression [7]. Therefore, anti-angiogenic therapies such as thalidomide and lenalidomide have emerged as essential therapeutic approaches to this disease [8-10]. Hitherto, although previous studies have shown that various signalling pathways such as HIF-1α, Notch1 and PI3K/Akt are involved in MM angiogenesis [11-13], the associated mechanisms have not been completely elucidated.

MicroRNAs (miRNAs or miRs) are small, non-coding, single-stranded RNAs (approximately 22 nucleotides long) that regulate protein levels by binding to either partial or complete complementary sites in messenger RNAs (mRNAs), thus leading to translational repression or transcript degradation, respectively [14]. Poliseno et al. [15] used miRNA microarrays to discover that 27 miRNAs were overexpressed in human umbilical vein ECs (HUVECs), 15 of which have been found to regulate angiogenic cytokine expression. In addition, more miRNAs with angiogenic roles have been identified. let-7 family member miRNAs have been shown to be pro-angiogenic and to promote tumour angiogenesis by inhibiting the anti-angiogenic factors thrombospondin-1 (TSP-1) and tissue inhibitor of metalloproteinase-1 (TIMP-1) [16,17]. miR-145 inhibits tumour growth and angiogenesis by targeting N-Ras and VEGF [18]. The miR-17/92 cluster (which encodes miR-17, −18a, −19a/b, −20a and −92-1) is overexpressed in tumour cells [19]. Previous studies revealed that the miR-17/92 cluster enhanced angiogenesis by downregulating the anti-angiogenic TSP1 and connective tissue growth factor (CTGF) [16,20]. Furthermore, this cluster was recently shown to exhibit anti-angiogenic activity via the inhibition of pro-angiogenic genes, including Janus kinase 1 (Jak1) [21] and the integrin subunit alpha 5 (ITG5a) [22].

Drosha and Dicer are 2 key RNase III enzymes that process pre-miRNAs into mature miRNAs, which are then incorporated into the RNA-induced silencing complex (RISC) to downregulate target gene activity by triggering either RNA degradation or translational repression [23]. Previous studies have reported that Dicer and Drosha act to drive angiogenesis both *in vitro* and *in vivo*[17,24,25]. In ECs, the downregulation of both enzymes decreased the capillary-sprouting and tubule-forming activities induced by regulatory miRNAs, including the let-7 family members and miR-27b [17]. The argonaute 2 (AGO2) protein is a core component of RISC [26]. AGO2 knockdown suppressed HUVEC growth and tube formation, suggesting that AGO2 also modulates angiogenesis [27,28]. Zhou et al. [29] reported that in MM patients, increased AGO2 expression was DNA copy number dependent and that AGO2 silencing could inhibit cell proliferation and promote apoptosis in myeloma cell lines. However, the mechanism by which AGO2 induces angiogenesis in MM has remained elusive. In the current study, we discovered that AGO2 can enhance MM angiogenesis *in vitro* and *in vivo*. Further studies revealed that angiogenic miRNAs are the key factors that promote this effect.

## Results

### AGO2 protein expression is associated with microvessel density (MVD) in MM patients

**Figure 1:**
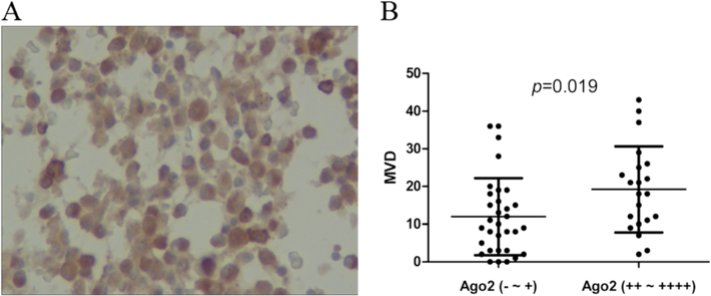
**AGO2 expression is associated with MVD in MM. (A)** Immunohistochemical analysis indicated AGO2 expression in the cytoplasm of myeloma cells from bone marrow biopsy samples. **(B)** The MVD was significantly higher in AGO2-high expression (++ to ++++) samples than in AGO2-low expression (− or +) samples (*p* = 0.019).

To investigate the relationship between the AGO2 protein expression levels and angiogenesis in MM, we detected the AGO2 protein levels in bone marrow biopsies from MM patients using an anti-AGO2 antibody and found that AGO2 protein localized in the myeloma cell cytoplasm (Figure 1A). AGO2-high expression (++ to ++++) was determined in 21 cases and AGO2-low expression (− or +) in 32 cases. MVD was used to evaluate angiogenesis in MM patients. The results showed that MVD was significantly higher in the AGO2-high expression samples than in the AGO2-low expression samples (19.24 ± 11.42 *vs.* 11.97 ± 10.20, *p* = 0.019; Figure 1B). An analysis of the correlation coefficients showed that AGO2 expression was also associated with MVD (r = 0.312, *p* = 0.023). The data suggested that AGO2 might enhance angiogenesis in MM patients.

### AGO2 accelerates HUVEC tube formation and migration in vitro

**Figure 2:**
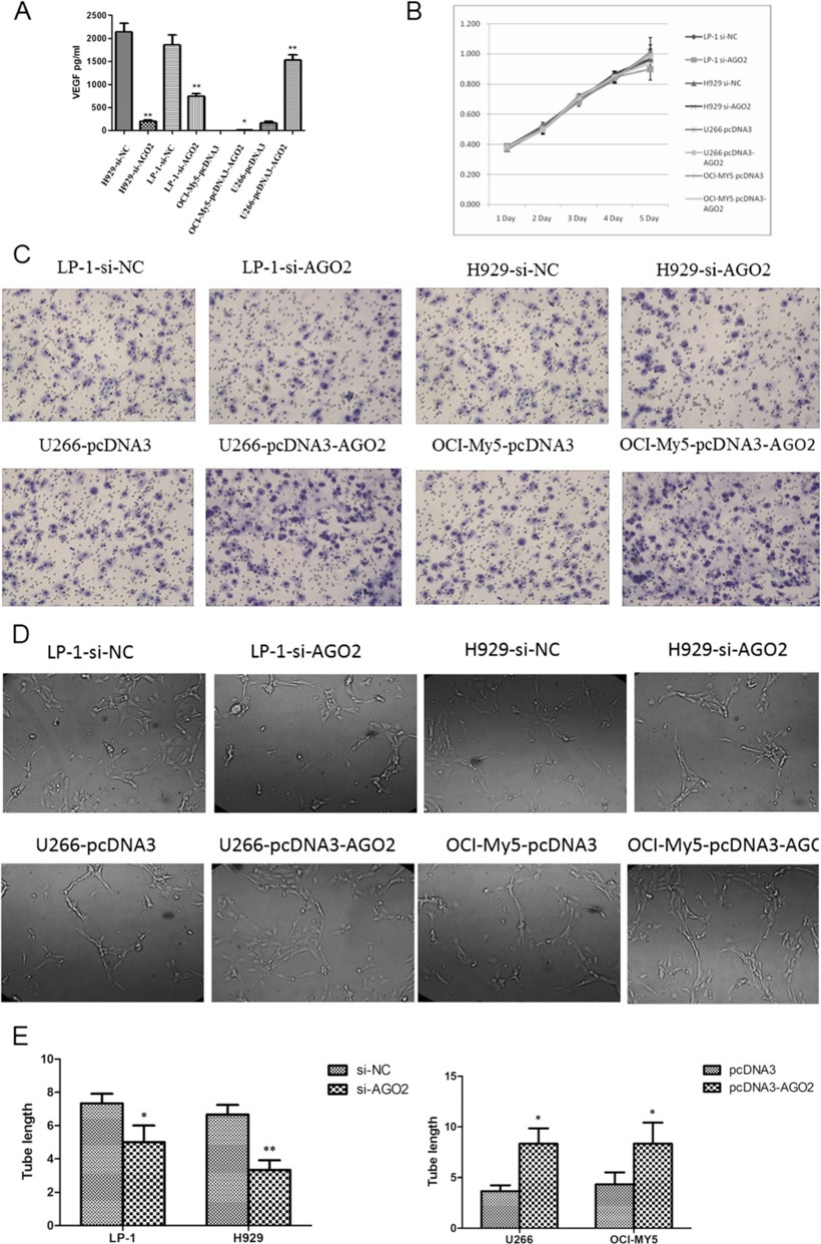
**AGO2 promotes myeloma angiogenesis. (A)** VEGF protein expression levels in the supernatants of AGO2-knockdown and overexpressing myeloma cell lines as determined in a LUMINEX cytokine assay (*p < 0.05, **p < 0.01). **(B)** HUVEC proliferation and viability in response to culture with the supernatants from AGO2-knockdown and overexpressing myeloma cell lines were evaluated in an MTT assay. Error bars represent the standard error of the mean for 2 independent experiments. **(C)** A Transwell migration assay showed that HUVEC migration decreased in cultures treated with supernatants from AGO2-knockdown cell lines (upper line) and increased in cultures with supernatants from AGO2-overexpressing myeloma cell lines (lower line). **(D,E)** HUVEC tube formation assay. Significant decreases in the tube number **(D)** and tube length **(E)** were observed in co-cultures with supernatants from AGO2-knockdown cell lines compared with those co-cultured with supernatants from scrambled control lines. Significant increases in the tube number **(D)** and tube length **(E)** were also observed in co-cultures with supernatants from AGO2-overexpressing MM cell lines compared with those co-cultured with supernatants from empty vector control lines (*p < 0.05, **p < 0.01).

To validate the pro-angiogenic functional role of AGO2 in MM, we used lentiviral transfection of an AGO2-shRNA to silence AGO2 expression in the H929 and LP-1 myeloma cell lines (H929-si-AGO2 and LP-1-si-AGO2) and also generated scramble controls (H929-si-NC and LP-1-si-NC; Additional file 1: Figure S1). We further established stable AGO2-overexpressing U266 and OCI-My5 myeloma cell lines (U266-pcDNA3-AGO2 and OCI-My5-pcDNA3-AGO2) and empty vector controls (U266-pcDNA3 and OCI-My5-pcDNA3; Additional file 1: Figure S1). We used a LUMINEX assay to test a number of angiogenesis-related cytokines such as matrix metalloproteinases 1, 2, 3, 7, 9, 10, 12 and 13 (MMP-1, 2, 3, 7, 9, 10, 12 and 13, respectively); FGF-2; PDGF-AA; PDGF-AB/BB; TIMP-1, 2, 3 and 4; granulocyte-macrophage colony stimulating factor (GM-CSF); VEGF and epidermal growth factor (EGF). The results revealed significantly elevated VEGF protein expression in the supernatants from the AGO2-overexpressing myeloma cell lines and decreased expression in the AGO2-knockdown cell lines (Figure 2A). No significant differences were observed in the MMP-9, FGF-2 and EGF expression levels (Table 1); the expression levels of the other cytokines were below the level of detection. As VEGF can mediate vascular permeability and induce EC growth and vasculogenesis, it thus plays crucial roles in MM angiogenesis, invasion and metastasis. Therefore, we further investigated the angiogenic activities of the supernatants from these AGO2-overexpressing or -knockdown cell lines.

**Table 1:** The expression of VEGF, MMP-9, EGF and FGF-2 as detected by LUMINEX

	**VEGF (pg/ml)**	**MMP-9 (pg/ml)**	**EGF (pg/ml)**	**FGF-2 (pg/ml)**
H929/Si-NC	2132.33 ± 328.02	15.03 ± 0.80	48.80 ± 10.42	98.30 ± 16.56
H929/SiR-AGO2	194.67 ± 54.12	14.00 ± 1.65	49.62 ± 4.22	94.86 ± 7.90
OCI-MY5/pcDNA3	0.92 ± 0.29	12.22 ± 1.48	55.96 ± 6.77	81.84 ± 9.04
OCI-MY5/pcDNA3-AGO2	10.36 ± 1.32	13.49 ± 1.80	53.74 ± 9.45	77.91 ± 5.98
LP-1/Si-NC	1858.00 ± 368.49	8.58 ± 0.85	147.34 ± 17.63	54.37 ± 9.14
LP-1/SiRNA-AGO2	748.56 ± 86.28	9.19 ± 0.85	146.77 ± 11.09	50.47 ± 7.19
U266/pcDNA3	164.97 ± 55.62	7.99 ± 1.40	177.94 ± 14.40	101.80 ± 13.62
U266/pcDNA3-AGO2	1522.00 ± 209.80	7.66 ± 1.27	173.05 ± 16.47	98.87 ± 14.38

Data presented as mean ± s.d. of triplicate samples.

VEGF Vascular endothelial growth factor; MMP-9 Matrix metalloproteinases; EGF Epidermal growth factor; FGF-2 Fibroblast growth factor-2.

To investigate the role of AGO2 in EC proliferation, the supernatants from these AGO2-overexpressing or -knockdown cell lines and relevant controls were added to HUVECs, and HUVEC proliferation was evaluated via the 3-(4,5-dimethylthiazol-2-yl)-2,5-diphenyltetrazolium bromide (MTT) method at 24, 48, 72, 96 and 120 h. No significant differences in HUVEC cell proliferation were observed in response to the supernatants from these cell lines (Figure 2B).

To assess the potential role of AGO2 activation in EC migration, a Transwell migration assay was performed to evaluate the ability of the HUVECs to migrate toward the supernatants from AGO2-knockdown or -overexpressing cell lines and relevant controls. The numbers of migrating HUVECs were 72.33 ± 10.26, 49.67 ± 4.73, 71.67 ± 6.51 and 53.33 ± 7.37 in response to the H929-si-NC, H929-si-AGO2, LP-1-si-NC and LP-1-si-AGO2 supernatants, respectively, and 70.33 ± 5.69, 95.67 ± 6.11, 66 ± 5.57 and 100 ± 9.165 in response to the U266-pcDNA3, U266-pcDNA3-AGO2, OCI-My5-pcDNA3 and OCI-My5-pcDNA3-AGO2 supernatants, respectively (Figure 2C). The results indicated that HUVEC migration decreased when the cells were cultured with supernatants from the H929 and LP-1 AGO2-knockdown cell lines and increased when the cells were cultured with supernatants from the AGO2-overexpressing U266 and OCI-My5 cell lines compared with the relevant controls (*p* = 0.044461, 0.03265, 0.006357 and 0.009216, respectively). The data revealed that AGO2 promotes HUVEC migration.

We also observed HUVEC tube formation *in vitro*. The results showed that the supernatants from the AGO2-knockdown H929 and LP-1 cell lines suppressed HUVEC tube formation at 72 h after incubation compared with that observed with supernatants from the relevant control cell lines. In contrast, supernatants from the AGO2-overexpressing U266 and OCI-My5 myeloma cell lines accelerated tube formation (Figure 2D). Additionally, the tube lengths were calculated using ImagePro Plus software to yield the following results: 7.33 ± 0.58, 6.67 ± 0.58, 3.67 ± 0.58 and 4.33 ± 1.15 μm/field with the LP-1-Si-NC, H929-Si-NC, U266-pcDNA3 and OCI-My5-pcDNA3 supernatants, respectively, and 5.33 ± 0.58, 3.33 ± 0.58, 8.33 ± 1.53 and 8.33 ± 2.08 μm/field with the LP-1-si-AGO2, H929-si-AGO2, U266-pcDNA3-AGO2 and OCI-My5-pcDNA3-AGO2 supernatants, respectively. The tube lengths decreased significantly when HUVECs were incubated with supernatants from H929-si-AGO2 and LP-1-si-AGO2 for 72 h and increased in response to supernatants from U266-pcDNA3-AGO2 and OCI-My5-pcDNA3-AGO2, relative to the respective controls (*p* < 0.05; Figure 2E). Taken together, all these data provide strong evidence that AGO2 can induce angiogenesis *in vitro*.

### AGO2 facilitates angiogenesis in vivo

**Figure 3:**
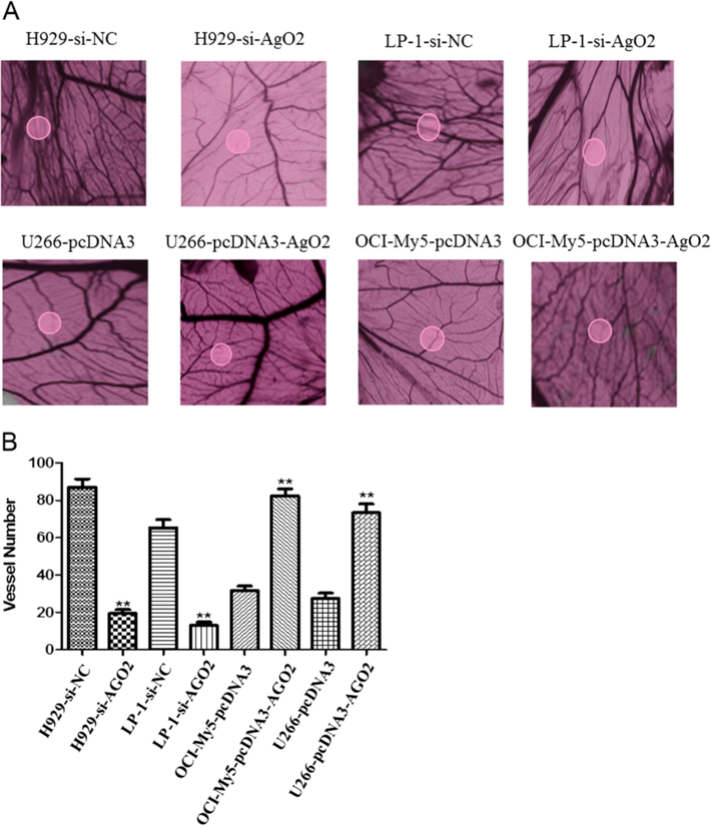
**Effects of the supernatants from AGO2-knockdown or -overexpressing MM cell lines on CAM neovascularization. (A)** The supernatants from AGO2-knockdown cell lines inhibited blood vessel formation, whereas those from AGO2-overexpressing cell lines facilitated blood vessel formation. **(B)** The number of blood vessels in each CAM (**p < 0.01).

A chick chorioallantoic membrane (CAM) assay was used to assess the effects of AGO2 on angiogenesis *in vivo*. Lower blood vessel densities were observed on the CAM surface at 4 days after infiltration with supernatants from the H929-si-AGO2 (19 ± 3) and LP-1-si-AGO2 (13 ± 3) cell lines, compared with infiltration with supernatants from the H929-si-NC (87 ± 7) and LP-1-si-NC (65 ± 8) control lines. In contrast, the numbers of infiltrating blood vessels were significantly higher in response to the supernatants from the U266-pcDNA3-AGO2 (73 ± 8) and OCI-My5-pcDNA3-AGO2 (82 ± 6) lines, compared with those from the U266-pcDNA3 (27 ± 5) and OCI-My5-pcDNA3 (32 ± 4) control lines (Figure 3A,B). These results further demonstrate the pro-angiogenic effects of AGO2 *in vivo*.

### AGO2 regulates a characteristic miRNA signature in MM

**Figure 4:**
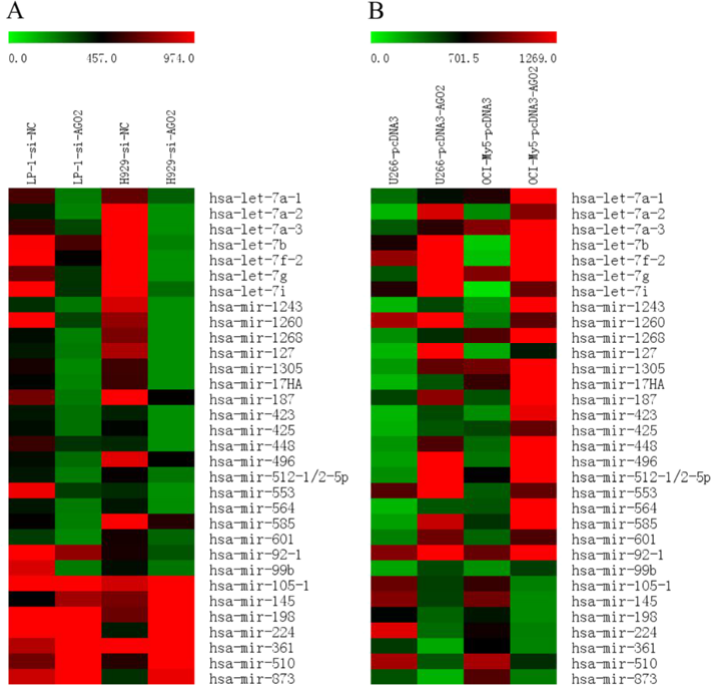
**AGO2-associated miRNA expression profiles. (A)** AGO2-regulated miRNAs in AGO2-overexpressing MM lines and **(B)** AGO2-regulated miRNAs in AGO2-knockdown MM lines.

AGO2 is a master regulator of mature miRNA genesis. To identify specific AGO2-regulated miRNA alterations, we determined the miRNA microarray profiles of the H929-si-AGO2, LP-1-si-AGO2, OCI-My5-pcDNA3-AGO2 and U266- pcDNA3-AGO2 lines and the respective controls. Our cut-off criteria for the miRNA expression analysis were as follows: upregulated genes, >1.5 and downregulated genes, <0.67. We first compared the miRNA microarray profiles of the H929-si-AGO2 and LP-1-si-AGO2 cell lines to those of the H929-si-NC and LP-1-si-NC lines, respectively. The analysis revealed that the expression levels of 51, 68, 120 and 97 miRNAs were increased and the levels of 199, 138, 104 and 69 miRNAs were decreased in the H929-si-AGO2, LP-1-si-AGO2, U266-pcDNA3-AGO2 and OCI-pcDNA3-AGO2 cell lines, respectively, relative to the levels in the respective controls. From these data, 100 miRNAs were down regulated and 8 miRNAs up regulated in the AGO2-knockdown cell lines whereas 17 miRNAs were down regulated and 74 miRNAs up regulated in the AGO2-overexpressing cell lines. Further analysis revealed that 25 miRNAs were commonly identified as upregulated and 7 miRNAs as downregulated by AGO2 (Figure 4A,B). In particular, many of these commonly dysregulated miRNAs are well-known angiogenic miRNAs, including the let-7 family members (let-7a-1, let-7a-2, let-7a-3, let-7b, let-7f-2, let-7 g and let-7i), 2 miR-17/92 cluster members (miR-17a and miR-92-1), miR-145 and miR-361.

### Validation of the AGO2-mediated dysregulation of angiogenic miRNA expression via quantitative reverse transcription–polymerase chain reaction (qRT-PCR)

We additionally performed qRT-PCR to analyse some of the above-mentioned dysregulated miRNAs and thus validate the microarray results. Consistent with the array data, let-7a-1, miR-17 and miR-92-1 expression decreased in the H929-si-AGO2 and LP-1-si-AGO2 cell lines and increased in the U266-pcDNA3-AGO2 and OCI-My5-pcDNA3-AGO2 cell lines compared with the respective controls (*p* < 0.05). Conversely, miR-145 expression was increased in the H929-si-AGO2 and LP-1-si-AGO2 cell lines and decreased in the U266-pcDNA3-AGO2 and OCI-My5-pcDNA3-AGO2 cell lines (Additional file 1: Figure S2). These data further confirmed our initial array results and supported the finding that these miRNAs were modulated by AGO2.

### miR-92-1 targets the angiopoietin-like protein 1 (ANGPTL1), an anti-angiogenic protein with tumour-inhibiting properties

**Figure 5:**
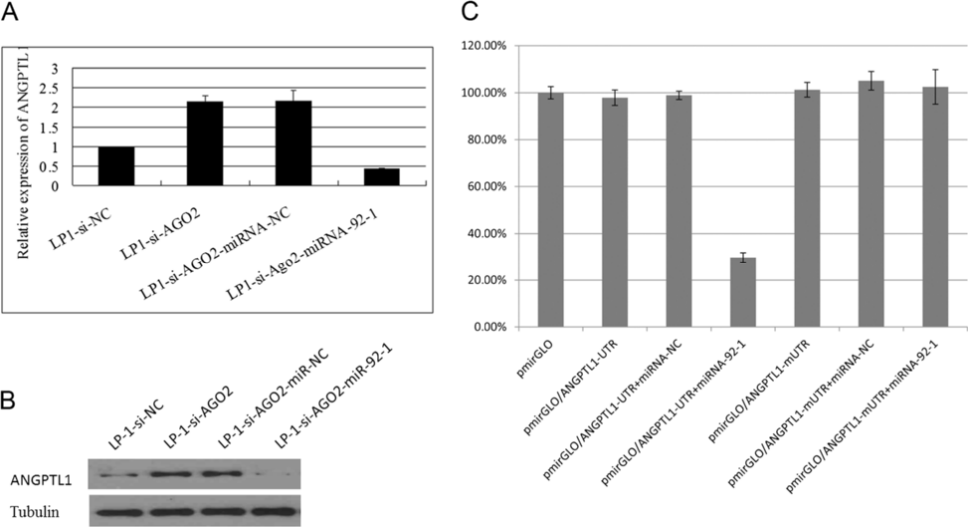
**miR-92-1 directly targets ANGPTL1.** The real-time PCR **(A)** and Western blot **(B)** detection of ANGPTL1 expression levels in LP-1-si-AGO2 cells after transfection with negative control and miR-92-1 mimics. **(C)** The effect of miR-92-1 on the luciferase activities of ANGPTL1 3′-UTR and ANGPTL1 3′-UTR with a mutated miR-92-1-binding site (mutant UTR, mUTR). miR-92-1 overexpression resulted in a significant decrease in the pmirGLO-reporter vector luciferase activity with ANGPTL1 3′-UTR but not with the vector containing the corresponding mutant.

Previous studies have identified an association between the critical miR-17/92 cluster genes and pathogenesis and poor prognosis in MM patients [30,31]. The miR-17/92 cluster targets the pro-apoptotic gene Bim to suppress MM apoptosis [30]. Our findings revealed that AGO2 increased miR-92-1 expression in MM and thus contributed to MM angiogenesis. Therefore, we used available target-prediction software modules (Target Scan, Pictar) to further identify miR-92-1 targets involved in myeloma angiogenesis. One such targets, ANGPTL1, was implicated in the negative regulation of angiogenesis [32]. To explore the relationship between ANGPTL1, miR-92-1and AGO2 in MM angiogenesis, we first analysed ANGPTL1 expression in the LP-1-si-AGO2 cell line and found that it was increased in this cell line compared with the LP-1-si-NC control line. Next we added either miR-92-1 mimics or miRNA-NC to the LP-1-si-AGO2 line and determined using qRT-PCR and Western blotting that ANGPTL1 protein expression was downregulated in the treated cells compared with the untreated LP-1-si-AGO2 cells (Figure 5A,B), suggesting that miR-92-1 mimics could abrogate the AGO2-knockdown-mediated overexpression of ANGPTL1. To prove that miR-92-1 regulated ANGPTL1 by interacting with the 3′-UTR of ANGPTL1, we cloned the ANGPTL1 3′-UTR and constructed a corresponding mutant in the predicted microRNA-binding site. After co-transfection with pmirGLO-reporter vectors and miR-92-1 mimics, the LP-1 cells transfected with miR-92-1 mimics exhibited a significant decline in luciferase activity compared with the miRNA-NC and mutated-ANGPTL-1 3′-UTR transfected cells (Figure 5C; *p* < 0.05), indicating that miR-92-1 could regulate ANGPTL1 expression at the transcriptional level by interacting with the 3′-UTR of ANGPTL1. These results demonstrated that ANGPTL1 was a direct target gene of miR-92-1 and suggested that the miR-92-1-repressing anti-angiogenic protein ANGPTL1 might contribute to AGO2-mediated myeloma angiogenesis.

### miR-145 targets the pro-angiogenic protein VEGF

**Figure 6:**
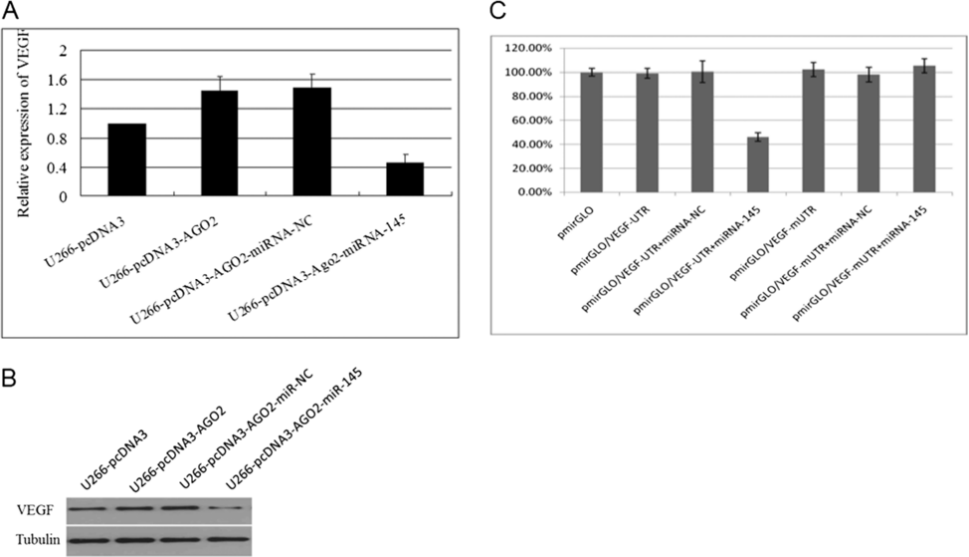
**miR-145 directly targets VEGF.** The real-time PCR **(A)** and Western blot **(B)** detection of VEGF expression levels in U266-pcDNA3-AGO2 cells after transfection with a negative control and miR-145 mimics. **(C)** The effect of miR-145 on the luciferase activities of VEGF 3′-UTR or VEGF 3′-UTR with a mutated miR-145-binding site (mUTR). miR-145 overexpression resulted in a significant decrease in the luciferase activity of the pmirGLO-reporter vector containing the VEGF 3′-UTR but not of the vector containing the corresponding mutant.

Previous studies have revealed that miR-145 inhibits tumour angiogenesis by directly targeting VEGF [18,33] in osteosarcoma and breast cancer. Our results also revealed that VEGF mRNA and protein expression levels in the miR-145 mimics-transfected U266-pcDNA3-AGO2 cell line were significantly decreased when compared with those in cells transfected with miRNA-NC mimics (Figure 6A,B). The luciferase activity level in U266 cells co-transfected with pmirGLO-VEGF-3′-UTR and miR-145 mimics was significantly decreased compared with that in cells co-transfected with miRNA-NC or mutated-VEGF-3′-UTR (Figure 6C). Therefore, the luciferase reporter assay also demonstrated that VEGF was a direct target gene of miR-145 in an MM cell line. All these data support a role for AGO2 in MM angiogenesis in which AGO2 mediates the miR-145-targeted inhibition of VEGF expression.

### let-7a targets HIF-3α, a regulator of VEGF

**Figure 7:**
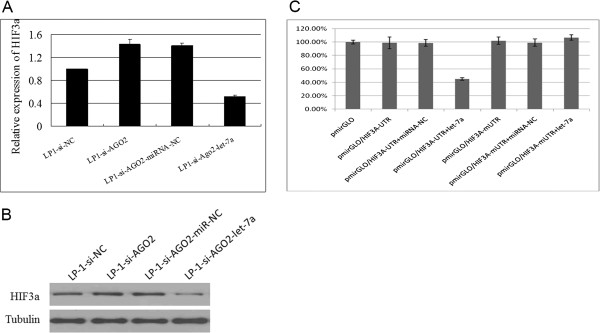
**AGO2-related let-7a targets HIF-3α.** The real-time PCR **(A)** and Western blot **(B)** detection of the HIF-3α expression levels in LP-1-si-AGO2 cells after transfection with a negative control and let-7a mimics. **(C)** The effect of let-7a on the luciferase activities of HIF-3α 3′-UTR or HIF-3α 3′-UTR with a mutated let-7a-binding site (mUTR). let-7a overexpression resulted in a significant decrease in the luciferase activity of the pmirGLO-reporter vector containing the HIF-3α 3′-UTR but not of the vector containing the corresponding mutant.

let-7 family members are known as pro-angiogenic miRNAs. Otsuka et al. [16] reported that TIMP-1 was a target of let-7b. However, the targets of the other let-7 family members are unclear. Herein, we found that the hypoxia-inducible factor (HIF)-3α (HIF-3α), a VEGF regulator, was a target of let-7a according to target-prediction software. qRT-PCR and Western blotting ascertained that HIF-3α expression was slightly elevated in the LP-1-si-AGO2 line but decreased again after the addition of let-7a mimics (Figure 7A,B). A luciferase reporter assay further demonstrated that let-7a mimics could inhibit the HIF-3α luciferase activity (Figure 7C). These results revealed that HIF-3α was a direct target gene of let-7a and therefore might mediate AGO2-induced MM angiogenesis.

## Discussion

In the current study, we investigated the role of AGO2 in myeloma angiogenesis. AGO2 can directly bind to miRNAs and thus mediate target mRNA degradation. Previous studies have validated the connection between AGO2 and early disease-related death in MM patients [34], and AGO2 silencing has been shown to inhibit cell viability in MM cell lines through decreased miR-106a, miR-106b, miR-17-5p and miR-20b expression and consequent further activation of the cyclin-dependent kinase inhibitors p21Waf1/Cip1 and p27Kip1 [29]. Our study, however, revealed a novel role and mechanism of AGO2 as an enhancer of myeloma angiogenesis through miRNA dysregulation, including the upregulation of pro-angiogenic miRNAs such as the let-7 family members and the miR-17/92 cluster and downregulation of the anti-angiogenic miRNA miR-145.

Hitherto, the role of AGO2 in tumour biogenesis has been unclear. AGO2 expression is elevated in colon cancers [35] and oestrogen receptor (ER) alpha-negative breast cancer cell lines [36]. In contrast, other studies have shown that AGO2 suppressed tumour growth and exhibited anti-cancer activity [37,38]. Asai et al. [27] first reported that AGO2 was required for angiogenesis. A robust body of evidence supports the importance of bone marrow angiogenesis in MM. Through EC activation, angiogenesis plays an essential role in tumourigenesis and represents a hallmark of MM progression [4,39]. In this study, we assessed the association between the AGO2 expression levels and MVD in MM and found a significant positive correlation between these factors. Furthermore, we studied the effects of AGO2 on HUVEC growth, migration and tube formation *in vitro*. Asai et al. [27] revealed that AGO2 silencing in HUVECs could suppress HUVEC proliferation and tube formation and trigger apoptosis. Although the present study did not find that the supernatants from AGO2-overexpressing MM lines affected HUVEC growth, it did reveal that supernatants from AGO2-overexpressing MM lines could induce HUVEC migration and accelerate tube formation. Conversely, the supernatants from AGO2-knockdown MM lines suppressed HUVEC cell migration and tube formation. Moreover, a CAM assay was used to demonstrate that supernatants from the AGO2-overexpressing MM lines could accelerate blood vessel formation, whereas supernatants from the AGO2-knockdown MM lines could inhibit blood vessel formation. Consistent with these findings, AGO2 exerts pro-angiogenic effects on MM both *in vitro* and *in vivo*.

AGO2 acts as a gatekeeper in RNA-silencing pathways and binds to an miRNA guide strand to mediate target mRNA cleavage or translational repression [40]. A previous study showed that elevation of the total miRNA expression levels in high-risk myeloma cells might be secondary to AGO2 dysregulation [29]. Using an miRNA microarray, we observed that 25 miRNAs were commonly upregulated and 7 miRNAs were commonly downregulated by AGO2. Of interest, the miRNAs regulated positively by AGO2 included most let-7 family members (let-7a-1, let-7a-2, let-7a-3, let-7b, let-7f-2, let-7 g and let-7i) and 2 miR-17/92 cluster members (miR-17a and miR-92-1), which are known pro-angiogenic miRNAs. Anti-angiogenic miRNAs such as miR-145 and miR-361 were negatively regulated by AGO2. Therefore, these miRNAs might contribute to AGO2-mediated MM angiogenesis.

Our study also provided important functional insights into some of the above-mentioned AGO-dysregulated angiogenic miRNAs. Previous studies have identified the miR-17/92 cluster, let-7 family and miR-145 as the modulators of angiogenesis [41]. The validated targets of the miR-17/92 cluster include TSP-1, CTGF, TIMP-1 and ITG5a. miR-92-1 can act through different targets as a pro-angiogenic or anti-angiogenic modulator in different diseases. We first discovered that ANGPTL1, a member of the angiopoietin-related protein family, was a target of miR-92-1 in MM cells. ANGPTL1 was identified as an anti-angiogenic factor that inhibits endothelial cell migration and tube formation [32,42]. Therefore, miR-92-1 might serve as a pro-angiogenic factor by suppressing ANGPTL1 expression in AGO2-mediated MM angiogenesis.

Previous studies have verified that VEGF plays a particularly important role in MM angiogenesis [43]. In this study, we found that AGO2-overexpressing MM lines facilitated VEGF protein secretion, whereas AGO2-knockdown MM lines suppressed VEGF protein secretion. Moreover, we observed that AGO2 concurrently inhibited miR-145 expression in MM. Decreased expression of miR-145, which acts as a tumour suppressor to inhibit the growth, angiopoiesis and migration of tumour cells, has been observed in several types of cancer [44-46]. VEGF has been identified as a key target of miR-145 during the inhibition of tumour angiogenesis. Herein, we confirmed that miR-145 could downregulate VEGF expression by directly binding to the 3′-UTR of VEGF, thus suggesting that AGO2 could accelerate VEGF secretion to promote blood vessel formation by inhibiting miR-145 expression in MM.

Additionally, HIF-1α and HIF-2α drive angiogenesis during tumour development [47], whereas HIF-3α negatively regulates the HIF pathway in vascular cells by decreasing hypoxia-mediated VEGF expression [48]. For the first time, we used a luciferase activity assay and Western blotting to validate that HIF-3α was truly a direct target of let-7a in MM. Therefore, we suggest that let-7a might promote myeloma angiogenesis by inhibiting its target (i.e. HIF-3α) and subsequently triggering VEGF expression. Taken together, these data suggest the strong likelihood that the dysregulation of the above-discussed AGO2-induced angiogenic miRNAs leads to AGO2-mediated angiogenesis in MM.

## Conclusions

The present study delineated the mechanistic roles of AGO2 in MM angiogenesis. AGO2 can drive MM neovessel formation *in vitro* and *in vivo* by dysregulating the expression of some angiogenic miRNAs. The pro-angiogenic let-7 family miRNAs, the miR-17/92 cluster and the anti-angiogenic miRNA miR-145 play crucial roles in AGO2-mediated angiogenesis by targeting angiogenesis-related genes.

## Methods

### Study subjects

This research was approved by the Hospital Review Board of the First Affiliated Hospital of Nanjing Medical University. All participants provided written informed consent in accordance with the Declaration of Helsinki. Bone marrow biopsy samples were obtained from 53 MM patients (33 males, 22 females) with a median age of 61.7 years (range, 38–80 years) who were recruited to this study between July 2010 and January 2013. MM was diagnosed according to standard morphological and immunophenotypical criteria. The monoclonal component was IgG in 18 cases, IgA in 12 cases, IgD in 1 case, IgM in 1 case, light chain in 19 cases and no secretion in 2 cases. According to the Durie–Salmon (DS) staging system, 5 patients were stage I, 5 were stage II and the remaining 43 were stage III. According to the International Staging System (ISS), 9 patients were stage I, 15 were stage II and the remaining 29 were stage III.

The MM cell lines (LP-1, H929, U266 and OCI-My5) were gifted from Dr Tian (University of Arkansas for Medical Science, USA) and cultured in RPMI-1640 media (Gibco, Grand Island, NY, USA). HUVECs were cultured in ECM media with 10% heat-inactivated foetal bovine serum (FBS, Gibco), 2-mM L-glutamine (Gibco), penicillin (100 U/mL) and streptomycin (100 μg/mL) at 37°C in a humidified chamber with 95% air and 5% carbon dioxide. HUVECs from passages 3–7 were used in all experiments.

### Immunohistochemical analysis and MVD assessment of bone marrow biopsies

Bone marrow biopsy samples were fixed in 10% formalin and decalcified in 10% nitric acid. Anti-CD138 was used to detect myeloma cells. An anti-AGO2 monoclonal antibody (EAU32; Novocastra Laboratories, Ltd., Newcastle-upon-Tyne, UK) was used to detect AGO2 expression in the myeloma cells from these samples. AGO2 staining was evaluated by 2 independent observers. The immunoreactive scores were determined according to the sum of the stained area and the intensity. Specifically, a score of 0 was assigned to a stained area with 0% reactivity, 1 for an area with >1% to <10% myeloma cells, 2 for >11% to <50% myeloma cells, 3 for >51% to <80% myeloma cells and 4 for >81% myeloma cells. For the staining intensity, a score of 0 was assigned for absent staining, 1 for weak staining, 2 for moderately intense staining and 3 for intense staining. The combined scores were recorded and graded as follows: −, 0–3; +, 4–5; ++, 6–8; +++, 9–10 and ++++, 11–12. Blood vessels were labelled with an anti-CD34 antibody (QBEnd10; Novocastra), which immunostained the EC. MVD was assessed by 2 independent observers. Three hot spots (the most intense microvasculature) were identified at 100× magnification, after which the microvessels (capillaries and venules) were counted at 400× magnification and the mean microvessels were calculated for the 3 hot spots. The mean count of the 2 independent quantifications was considered the final measurement for each hot spot.

### AGO2 gene overexpression or knockdown in MM cell lines

Synthetic double-stranded oligonucleotide sequences encoding the AGO2-shRNA and scramble control siRNA were described previously [29]; these were cloned into lentiviral pSRL-SIH1 vectors. Recombinant lentivirus was produced by transfecting 293 T cells according to a standard protocol. Lentiviral AGO2 shRNA and scramble (SCR) shRNA were produced in 293 T packaging cells, concentrated at a multiplicity of infection (MOI) of 50 and individually added to H929 and LP-1 cell suspensions in the presence of 5 g/mL of polybrene. All transfection experiments were performed in duplicate.

The amplified 2580-bp AGO2 cDNA sequence (forward primer: 5′-CGCGAATTCATGTAC TCGGGAGCCGGCC-3′, reverse primer: 5′-GCTCTAGATCAAGCAAAGTACATGGTG-3′) was cloned into the pcDNA3 vector to construct the pcDNA3-AGO2 expression plasmid. pcDNA3-AGO2 and pcDNA3 empty vector (EV) plasmids were transfected into U266 and OCI-My5 cell lines using Lipofectamine 2000 (Invitrogen, Carlsbad, CA, USA); transfections were performed in 6-well plates. After a 72-h incubation, G418 (Invitrogen; 400 μg/ml) was used to select G418-resistant cells. Stable AGO2-overexpressing U266 and OCI-My5 clones were established after 3 months. Stable EV clones of the 2 cell lines were also constructed and served as controls.

### Cell proliferation assay

Pancreatin-digested HUVECs were added to 96-well plates at a density of 5 × 10^3^ cells/well and were incubated with supernatants from AGO2-knockdown or -overexpressing myeloma cells. An MTT colorimetric assay was performed to evaluate the cell viability at 24, 48, 72, 96 and 120 h. Twenty microlitres of MTT (5 mg/ml) were added to each well. After a 4-h incubation, the supernatant fluid was discarded and 100 μl of DMSO were added to each well. The absorbance intensity was measured at 490 nm with a microplate reader (BioTek, Winooski, VT, USA).

### Transwell migration assay

To evaluate EC migration, a 24-well Transwell plate (Corning Costar, Corning, NY, USA) with an 8.5-μm polycarbonate membrane was used. The undersurface of the Transwell was coated with 10 μg/ml of collagen I (Sigma, St. Louis, MO, USA). HUVECs were seeded in the upper chambers in 100 μl of 10% FBS RPMI-1640 medium; 600 μl of supernatants from AGO2-knockdown or -overexpressing myeloma cells were added to the lower chambers. SCR shRNA and pcDNA3-EV-transfected myeloma cell line supernatants were used as controls. After a 24-h incubation at 37°C and 5% CO_2_, the non-migrating HUVECs on the upper sides of the membranes were removed, and the migrated cells on the lower sides of the membranes were stained with crystal violet and counted under an Olympus optical microscope (Olympus Corporation, Tokyo, Japan).

### Tube formation assay

The HUVEC tube formation assay was performed according to the manufacturer’s instructions. Trypsinized HUVECs (1 × 10^4^) were seeded onto a Matrigel-coated (BD Biosciences, San Jose, CA, USA) 24-well plate and incubated with cell-free culture supernatants from AGO2-knockdown or -overexpressing myeloma cells and controls at 37°C and 5% CO_2_ for 72 h. The degree of tube formation was evaluated using an inverted microscope. The numbers of tubes were calculated using ImagePro Plus software (Media Cybernetics Inc., Rockville, MD, USA).

### CAM assay

A CAM assay [49] was performed to determine the angiogenic activity of AGO2 *in vivo*. Fertilized 7-day-old chicken eggs were obtained from a local hatchery. A small (0.6 × 1.0 cm) window was made on each shell, and a 0.5-cm diameter sterile filter paper soaked with AGO2-knockdown or -overexpressing myeloma cell supernatant was loaded onto each CAM. The windows were then sealed with sterile tape and the eggs were incubated at 37.5°C. Following an additional 96-h incubation, images of each treated CAM were captured under a dissecting microscope, and the blood vessels in 5 filter paper fields were counted at 40× magnification to evaluate angiogenesis.

### miRNA microarray data analysis

Total RNA was extracted from the myeloma cell lines using TRIzol (Invitrogen). A total of 640 DNA oligonucleotide probes from the mirVana miRNA Probe Set (Ambion/Life Technologies, Carlsbad, CA, USA) were designed according to the sequences of their respective mature miRNAs. The probes were resuspended at a concentration of 50 mM in 3× saline sodium citrate (SSC) and spotted onto MICROMAX SuperChip I Glass Slides (PerkinElmer Inc., Waltham, MA, USA) in duplicates at 50%–60% humidity, using a SpotArray 24 Microarray Printing System (PerkinElmer). Small RNAs were labelled with Cy5 or Cy3 dyes (Amersham Biosciences, Piscataway, NJ, USA) using the mirVana miRNA Labelling Kit (Ambion/Life Technologies). After an overnight (12–16 h) hybridization at 42°C, the slides were washed in SSC and scanned with a ScanArray Express Microarray Scanner and ScanArray 3.0 software (PerkinElmer). A >1.5-fold increase or a <0.67-fold decrease in the expression level was set as the threshold to indicate a significant change.

### miRNA quantitative RT-PCR (qPCR) analysis

miRNA qRT-PCR was performed with the SYBR Premix Ex TaqTM kit according to the manufacturer’s recommendations, and qRT-PCR was performed on an iQ5 real-time PCR detection system (Bio-Rad Laboratories Inc., Hercules, CA, USA). The relative gene expression levels were calculated according to the 2^−ΔΔCt^ method. All primers were purchased from Genewiz, Inc. (Suzhou, China). Each sample was normalized to the mean U6 expression value. Comparative real-time PCR was performed in triplicate throughout the study.

### miRNA oligonucleotide mimics transfection experiments

miRNA oligonucleotide mimics were designed and synthesized by GenePharma (Shanghai, P.R. China) and transfected into LP-1 cell lines using Lipofectamine 2000 (Invitrogen). The mimic sequences were as follows: miRNA-negative control (miRNA-NC) mimics, UUCUCCGAACGUGUCACGUTT, ACGUGACACGUUCGGAGAATT; hsa-mir-92-1 mimics, AGGUUGGGAUCGGUUGCAAUGCU, AGCAUUGCAACCGAUCCCAACCU; hsa-let-7a mimics, UGAGGUAGUAGGUUGUAUAGUU, AACUAUACAACCUACUACCUCA and hsa-mir-145 mimics, GUCCAGUUUUCCCAGGAAUCCCU, AGGGAUUCCUGGGAAAACUGGAC. Each experiment was performed in triplicate.

### Luciferase reporter assay

The ANGPTL1-3′-UTR, HIF-3α-3′-UTR and VEGF-3′-UTR sequences and corresponding mutated sequences were cloned into the pmirGLO Dual-Luciferase miRNA Target Expression Vector. LP-1 or U266 myeloma cells were co-transfected with the different cloned pmirGLO (0.2 μg) plasmids and miRNA mimics (10 pmol) using Lipofectamine 2000 (Invitrogen). The cells were harvested after 48 h and analysed with the Dual Luciferase Reporter Assay kit (Promega, Madison, WI, USA) according to the manufacturer’s recommendations. Firefly and Renilla luciferase activities were quantified in the cell lysates. The luciferase readings were corrected for background, and the firefly luciferase values were normalized to the Renilla values to control for transfection efficiency. All assays were performed in triplicate.

### Western blot analysis

Western blots were performed as previously described [19]. Briefly, cells were lysed with a lysis buffer for 30 min on ice. Fifty micrograms of protein were fractionated via 10% SDS-PAGE and transferred onto nitrocellulose membranes (EMD Millipore, Billerica, MA, USA). The membranes were then probed with appropriate primary antibodies (1:500 dilution), including anti-AGO2, anti-VEGF, anti-ANGPTL1, anti-HIF-3α and anti-tubulin antibodies (Abcam, Cambridge, UK), followed by incubation with HRP-conjugated secondary antibodies (1:5000 dilution). The probed proteins were then detected with an enhanced chemiluminescent substrate (NEL100001EA; PerkinElmer). The tubulin expression level served as an internal control.

### LUMINEX cytokine assay

The LUMINEX assay was performed on a Luminex FLEX MAP 3D platform with the MILLIPLEX MAP Human Cytokine/Chemokine Panel (Millipore, USA) according to the manufacturer’s instructions. We detected a variety of cytokines (MMP-1, MMP-2, MMP-3, MMP-7, MMP-9, MMP-10, MMP-12, MMP −13, FGF-2, PDGF-AA, PDGF-AB/BB, TIMP-1, TIMP-2, TIMP-3, TIMP-4, GM-CSF, VEGF and EGF). The analysis of cytokine expression in the supernatants from the AGO2-knockdown and overexpressing myeloma cell lines was also performed in triplicate; all values were calculated via extrapolation from the standard curve.

### Statistical analysis

The statistical analysis was performed using GraphPad Prism 5 software (GraphPad, Inc., San Diego, CA, USA). The numerical results are expressed as means ± standard deviations. The differences in the results among the groups were statistically analysed using the unpaired Student's t test and the Mann–Whitney U-test. Correlations of AGO2 with MVD were performed using Spearman’s correlation. *P* values of <0.05 were considered statistically significant.

## Abbreviations

miRNA: MicroRNA; MM: Multiple myeloma; AGO2: Argonaute 2; RISC: RNA-induced silencing complex; mRNA: Messenger RNA; HUVEC: Human umbilical vein endothelial cell; VEGF: Vascular endothelial growth factor; PDGF: Platelet-derived growth factor; FGF: Fibroblast growth factor; TSP-1: Thrombospondin-1; TIMP-1: Tissue inhibitor of metalloproteinase-1; CTGF: Connective tissue growth factor; ITG5a: Integrin subunit alpha5; MVD: Microvessel density; MMP-1: Matrix metalloproteinases-1; MMP-2: Matrix metalloproteinases-2; MMP-3: Matrix metalloproteinases-3; MMP-7: Matrix metalloproteinases-7; MMP-9: Matrix metalloproteinases-9; MMP-10: Matrix metalloproteinases-10; MMP-12: Matrix metalloproteinases-12; MMP-13: Matrix metalloproteinases-13; FGF-2: Fibroblast growth factor-2; PDGF-AA: Platelet-derived growth factor-AA; PDGF-AB/BB: Platelet-derived growth factor-AB/BB; TIMP-1: Tissue inhibitor of matrix metalloproteinases-1; TIMP-2: Tissue inhibitor of matrix metalloproteinases-2; TIMP-3: Tissue inhibitor of matrix metalloproteinases-3; TIMP-4: Tissue inhibitor of matrix metalloproteinases-4; M-CSF: Granulocyte-macrophage colony stimulating factor; VEGF: Vascular endothelial growth factor; EGF: Epidermal growth factor; MTT: 3-(4,5-dimethylthiazol-2-yl)-2,5-diphenyltetrazolium bromide; CAM: Chick chorioallantoic membrane; qRT-PCR: Quantitative reverse transcription–polymerase chain reaction; ANGPTL1: Angiopoietin-like protein 1; HIF-3α: HIF-3α; ER: Oestrogen receptor; DS: Durie–salmon; ISS: International staging system; SCR: Cramble; MOI: Multiplicity of infection; EV: Empty vector; SSC: Saline sodium citrate; QPCR: Quantitative RT-PCR.

## Competing interests

The authors declare that they have no competing interests.

## Authors’ contributions

SW and WJY contributed equally to this work. SW and WJY performed the research, analyzed and interpreted the data; RW did the immunohistochemical analysis and histologic assessment; JX, XYQ, JRX, QGZ and JYL designed the research and contributed to clinical samples. LJC designed the research, analyzed the clinical data and drafted the manuscript. All authors read and approved the final manuscript.

## Supplementary Material

Additional file 1: Figure S1 AGO2 protein expression in AGO2-knockdown and -overexpression myeloma cell lines. **Figure S2.** The relative expression of AGO2-regulated miRNAs in AGO2 knockdown or over-expression myeloma cell lines by RT- PCR. **(A)** miR-17 expression; **(B)** miR-92-1 expression; **(C)** miR-145 expression; **(D)** Let-7a expression. **Table S1.** The normal and mutated sequence of predicted miRNAs-binding site in target gene 3’UTR. Click here for file
