# Supplementary material for: Argonaute 2 promotes myeloma angiogenesis via microRNA dysregulation
Source: J Hematol Oncol. 2014 May 7;7:40. doi: 10.1186/1756-8722-7-40 (PMC4108130; doi:10.1186/1756-8722-7-40)
Supplement: Additional file 1: Figure S1 — AGO2 protein expression in AGO2-knockdown and -overexpression myeloma cell lines. Figure S2. The relative expression of AGO2-regulated miRNAs in AGO2 knockdown or over-expression myeloma cell lines by RT- PCR. (A) miR-17 expression; (B) miR-92-1 expression; (C) miR-145 expression; (D) Let-7a expression. Table S1. The normal and mutated sequence of predicted miRNAs-binding site in target gene 3’UTR. [file 1756-8722-7-40-S1.doc]

**Figure S1**


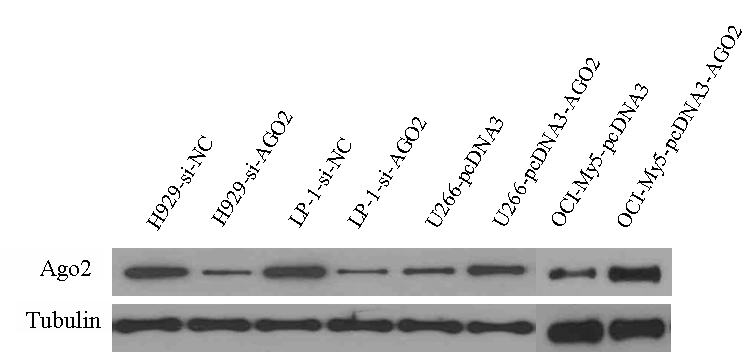


**Figure S1. AGO2 protein expression in AGO2-knockdown and -overexpression myeloma cell lines.**

**Figure S2**

**Figure S2. The relative expression of AGO2-regulated miRNAs in AGO2 knockdown or over-expression myeloma cell lines by RT- PCR.(A) miR-17 expression; (B)miR-92-1 expression; (C) miR-145 expression; (D)Let-7a expression.**

**Table S1**

**Table S1. The normal and mutated sequence of predicted miRNAs-binding site in target gene 3’UTR**
